# Construction and Application of an Electrochemical Sensor for Determination of D-Penicillamine Based on Modified Carbon Paste Electrode

**DOI:** 10.3390/mi15020220

**Published:** 2024-01-31

**Authors:** Arefeh Mohammadnavaz, Hadi Beitollahi, Sina Modiri

**Affiliations:** 1 Department of Chemistry, Graduate University of Advanced Technology, Kerman 76311-33131, Iran; 2 Environment Department, Institute of Science and High Technology and Environmental Sciences, Graduate University of Advanced Technology, Kerman 76311-33131, Iran; 3 Polymer Department, Graduate University of Advanced Technology, Kerman 76311-33131, Iran; sina.modiri@gmail.com

**Keywords:** electrocatalytic mechanism, multi-walled carbon nanotubes, Co_3_O_4_ nanoparticles, ionic liquid, benzoyl-ferrocene, carbon paste electrode, D-penicillamine

## Abstract

D-penicillamine (D-PA) is a sulfur-containing drug that has been used for various health conditions. However, like any medication, overdosing on D-PA can have adverse effects and may require additional treatment. Therefore, developing simple and sensitive methods for sensing D-PA can play a crucial role in improving its efficacy and reducing its side effects. Sensing technologies, such as electrochemical sensors, can enable accurate and real-time measurement of D-PA concentrations. In this work, we developed a novel electrochemical sensor for detecting D-PA by modifying a carbon paste electrode (CPE) with a multi-walled carbon nanotube-Co_3_O_4_ nanocomposite, benzoyl-ferrocene (BF), and ionic liquid (IL) (MWCNT-Co_3_O_4_/BF/ILCPE). Cyclic voltammetry (CV), differential pulse voltammetry (DPV), and chronoamperometry (CHA) were employed to explore the electrochemical response of D-PA on the developed sensor, the results of which verified a commendable electrochemical performance towards D-PA. Under optimized conditions, the developed sensor demonstrated a rapid response to D-PA with a linear dynamic range of 0.05 μM–100.0 μM, a low detection limit of 0.015 μM, and a considerable sensitivity of 0.179 μA μM^−1^. Also, the repeatability, stability, and reproducibility of the MWCNT-Co_3_O_4_/BF/ILCPE sensor were studied and showed good characteristics. In addition, the detection of D-PA in pharmaceutical and biological matrices yielded satisfactory recoveries and relative standard deviation (RSD) values.

## 1. Introduction

Today, drug analysis is a crucial aspect of scientific research. Drugs are highly diverse compounds with varying chemical structures and properties. The chemical structure of a drug specifies its physicochemical properties as well as its absorption, distribution, and metabolism while also influencing its pharmacological activity and effectiveness. The effectiveness and performance of these compounds are highly dependent on dosage. Each drug has a therapeutic range; the presence of the drug in lower concentrations has weak effects on patients, while higher concentrations cause side effects that can be dangerous for patients. Therefore, determining trace amounts of drugs in pharmaceutical compounds and biological fluids including plasma, blood serum, and urine is one of the ways to prevent side effects and increase the therapeutic properties of drugs, and it has a great impact on public health. In addition, drug analysis has a significant role in the drug development process by ensuring the safety, quality, and efficacy of new drugs.

The hydrolytic degradation of penicillin leads to the formation of penicillamine (PA), a potent chelating agent that has various therapeutic applications and reacts with diverse heavy metals. PA is of great significance in the pharmaceutical field because of its appreciable metal-binding capability [1,2,3]. Penicillamine is an amino acid containing a thiol group that exists in both D and L enantiomeric forms, each with distinct biological and toxicological properties. D-penicillamine is the pharmaceutical form of penicillamine but the L-penicillamine form is toxic as it inhibits the action of pyridoxine. D-PA is extensively prescribed to treat Wilson’s disease (hepatolenticular degeneration) and some other conditions such as primary biliary cirrhosis, rheumatoid arthritis, progressive systemic sclerosis, fibrotic lung diseases, scleroderma, heavy element poisoning, and cystinuria [4,5,6]. It inhibits the activity of macrophages, which in turn lowers the T-lymphocyte count, inhibits collagen from cross-linking, and reduces rheumatoid factor. Routine dosage of this drug for humans ranges from 0.5 to 2 g/day. Despite the positive effects of D-PA, its excessive use can be associated with serious complications like anorexia, oral ulceration, nephrotic syndrome, loss of taste, hematological problems, skin rashes, and glomerulonephritis, especially nephrotic syndrome [7,8,9,10]. In this respect, it is important to design and develop a quick, accurate, sensitive, and low-cost analytical method for the determination of D-PA.

There have been various techniques for the detection of D-PA in various matrices, some of which are high-performance liquid chromatography with electrochemical (HPLC-EL) detection [11], HPLC with fluorescence (HPLC-FL) detection and capillary electrophoresis with laser-induced fluorescence (CE-LIF) detection [12], chemiluminescence detection [9], colorimetric detection [13], fluorescence detection [14,15], and electrochemical detection [5,16,17,18,19,20,21,22,23,24]. However, some of these techniques suffer from some shortcomings, i.e., high cost, time consumption, and difficult pretreatment.

Electrochemical approaches have attracted further attention because they are highly sensitive, highly selective, rapid, affordable, and easy to use [25,26,27]. In recent years, various electrochemical sensors have been developed for analyte detection. However, high potentials are needed for electro-oxidation of diverse analytes on bare electrodes, with slow kinetics. In electrochemistry, modifying electrode surfaces is a key phase that could potentially solve or eliminate the mentioned bottlenecks [28,29,30,31,32]. 

Carbon-based electrodes like carbon paste electrodes (CPEs) with chemical inertness, simplified construction, rapid surface renewal, broad potential window, and affordability have been extensively exploited as diverse pharmaceutical and biological species sensor. The surface of CPE can be modified chemically by adding various materials to improve selectivity, rapidity, and sensitivity [33,34,35]. The concurrent modification of electrodes using ionic liquid, nanomaterials, and other conductive mediators introduces novel means to measure selective pharmaceutical formulations [36,37,38,39]. 

Nanotechnology is a rapidly developing scientific field that involves manipulating, controlling, and reforming materials at distinctive levels to effectuate new properties and capacities that generate interesting applications. Rapid progress in nanotechnologies has led to the wide application of this technology in several fields like medicine, catalysis, and energy [40,41,42,43,44,45]. Reducing the size of materials to the nanometer scale increases surface area and leads to new developments like great electro-conductivity [46,47,48]. Metal oxide nanoparticles are popular because their particle size, crystallite size, morphology, and crystalline phase can control their physicochemical features [49]. The popularity of cobalt oxide nanoparticles (Co_3_O_4_ NPs) can be attributed to their biocompatibility, large surface area, chemical stability, green nature, admirable conductivity, electronic profile, availability, electrical catalytic performance, antifouling capacity, and affordability [50]. Carbon nanomaterials have been widely studied as an emerging class of materials and have attracted attention across many different fields for their excellent electrical, optical, thermal, mechanical, and chemical properties and versatile applications. Since the discovery of carbon nanotubes (CNTs) by Iijima (1991), they have gained popularity in chemical, physical, and materials fields due to their unique structural, chemical, mechanical, and electronic properties [51]. Such unparalleled features, along with the use of catalyst support for size and dispersion control, accelerated electron transfer, strong electrocatalytic performance, great thermal conductivity, appreciable biocompatibility, and excellent interfacial adsorption features have elevated CNTs as semiconductor materials for the fabrication of electrochemical sensors [52,53]. Following chemical functionalization, multi-walled carbon nanotubes (MWCNTs) possess commendable adsorptive ability, catalytic performance, and electron transfer, thereby making them a promising platform for metals and metal nanoparticles. The CNTs/metal oxide nanoparticle composites offer great electrochemical responses for electrochemical sensors because of the best aspect ratio, biocompatibility, and electro-conductivity [54,55].

Ferrocene (Fc) and relevant derivatives have spurred extensive interest in the field of electroanalysis owing to their commendable redox behavior. Fc derivatives are attractive electrochemically active materials and the redox reaction of Fc^+^/Fc is completely reversible, so it has been employed in the construction of chemically modified electrodes [56,57,58]. Ionic liquids (ILs) are salts that are in a liquid state below 100 °C and are composed of organic cations and (in)organic anions. They have a nearly unlimited range of structural diversity and physicochemical properties that can be altered through the appropriate selection and modification of cations and anions. Over the past few decades, ILs have gained significant attention due to their unique characteristics and have become adaptable and novel materials for various applications. They are being used in multiple fields such as catalysis, material synthesis, photoelectric transformation, separation, and energy storage. More specifically, due to their unique properties, ILs have been used in many electrochemical applications, including electrocatalysts, electrochemical deposition, electrochemical equipment, and sensors. ILs have interesting features such as high thermal stability, high conductivity, and greater solubility than other electrolytes. Their non-flammability, low volatility, and also their electrochemical and thermal stability have made them suitable for making electrochemical sensors [59]. In general, the most characteristic features of ILs for use in the fabrication of electrochemical sensors and biosensors include a wide electrochemical window and high electrical conductivity.

Considering this, in this work, we demonstrate a sensitive D-PA electrochemical sensor through the synergetic effect of a MWCNT-Co_3_O_4_ nanocomposite, benzoyl-ferrocene (BF), and IL. The modified CPE provides high electrochemical performance towards D-PA. The proposed MWCNT-Co_3_O_4_/BF/ILCPE sensor exhibits the characteristic properties of individual components toward the oxidation of D-PA, the results of which highlighted acceptable sensitivity towards D-PA in real specimens. The novelty of the presented research is the application of the MWCNT-Co_3_O_4_ nanocomposite, BF, and IL as modifier species for the modification of CPE and its utilization for D-PA determination. Also, it should be noted that, to the best of our knowledge, no attempts have been made to investigate the applications of MWCNT-Co_3_O_4_/BF/ILCPEs as an electrochemical sensing platform for D-PA.

## 2. Experimental Design

### 2.1. Equipment and Materials

Electrochemical techniques such as CV, DPV, and CHA were carried out at ambient temperature with an Autolab potentiostat/galvanostat (PGSTAT302N, EcoChemie, Utrecht, The Netherlands), controlled by GPES 4.9 software. The techniques were performed using a three-electrode electrochemical cell consisting of three electrodes: (a) a working electrode (MWCNT-Co_3_O_4_/BF/ILCPE and other CPEs), (b) a reference electrode (Ag/AgCl/KCl (3.0 M)), and (c) an auxiliary or counter electrode (platinum wire). A pH meter (Metrohm type 713, Herisau, Switzerland) was applied to accurately measure the pH of different solutions. All solvents and chemicals were of analytical grade and used without further purification.

The synthesis and characterization of the MWCNT-Co_3_O_4_ nanocomposite was reported in our previous work [60].

### 2.2. Preparation of MWCNT-Co_3_O_4_/BF/ILCPE

To obtain the best conditions in the preparation of the MWCNT-Co_3_O_4_/BF/ILCPEs, we optimized the ratio of BF, IL, and MWCNT-Co_3_O_4_. Our results show that the maximum peak current intensity of D-PA could be obtained at the surface of the MWCNT-Co_3_O_4_/BF/ILCPE with an optimum ratio of BF, IL, and MWCNT-Co_3_O_4_.

For the preparation of the MWCNT-Co_3_O_4_/BF/ILCPE, appropriate amounts of BF (0.01 g), graphite powder (0.94 g), and MWCNT-Co_3_O_4_ nanocomposite (0.05 g) were hand-mixed. Then, paraffin (0.6 mL) and IL (0.2 mL) were added in the resulting mixture, followed by hand-mixing well for at least 30 min to prepare a homogeneous paste. In the next step, the prepared homogeneous carbon paste was packed into a glass tube. A copper wire was inserted into the carbon paste for electrical contact. After fabrication, the surface of the modified CPE was polished on weighing paper and cleansed using deionized water.

Moreover, the preparation of unmodified CPE (in the absence of IL, BF, and MWCNT-Co_3_O_4_ nanocomposite), MWCNT-Co_3_O_4_/CPE (in the absence of IL and BF), BF/ILCPE (in the absence of MWCNTs/Co_3_O_4_ nanocomposite), and MWCNT-Co_3_O_4_/ILCPE (in the absence of BF) was performed similar to the preparation of MWCNT-Co_3_O_4_/BF/ILCPE.

### 2.3. Preparation of Pharmaceutical (D-PA Capsules) and Biological (Urine) Samples

For the preparation of the sample solution for determining D-PA in pharmaceutical formulations, the contents of five capsules (D-PA = 250 mg/capsule) were completely grounded and homogenized to a fine powder using mortar and pestle. Then, a portion of the powder corresponding to the weight of the contents of one capsule was weighed and dissolved in a certain amount of deionized water by ultrasonication (30 min). After complete dissolution, this solution was filtered through a filter paper. Next, the resulting supernatant was collected and diluted with PBS (pH = 7.0). Finally, the prepared solution was input into the electrochemical cell and used for D-PA determination by the standard addition method.

Urine samples obtained from healthy volunteers were centrifuged at 2000 rpm for 10 min. Then, the collected supernatant was filtered using a paper filter (0.45 µm) and diluted with 0.1 M PBS (pH = 7.0). The prepared sample was used for analysis by spiking the known concentration of D-PA.

## 3. Results and Discussion

### 3.1. Evaluation of the Electrocatalytic Activity of MWCNT-Co_3_O_4_/BF/ILCPE towards D-PA 

**Figure 1:**
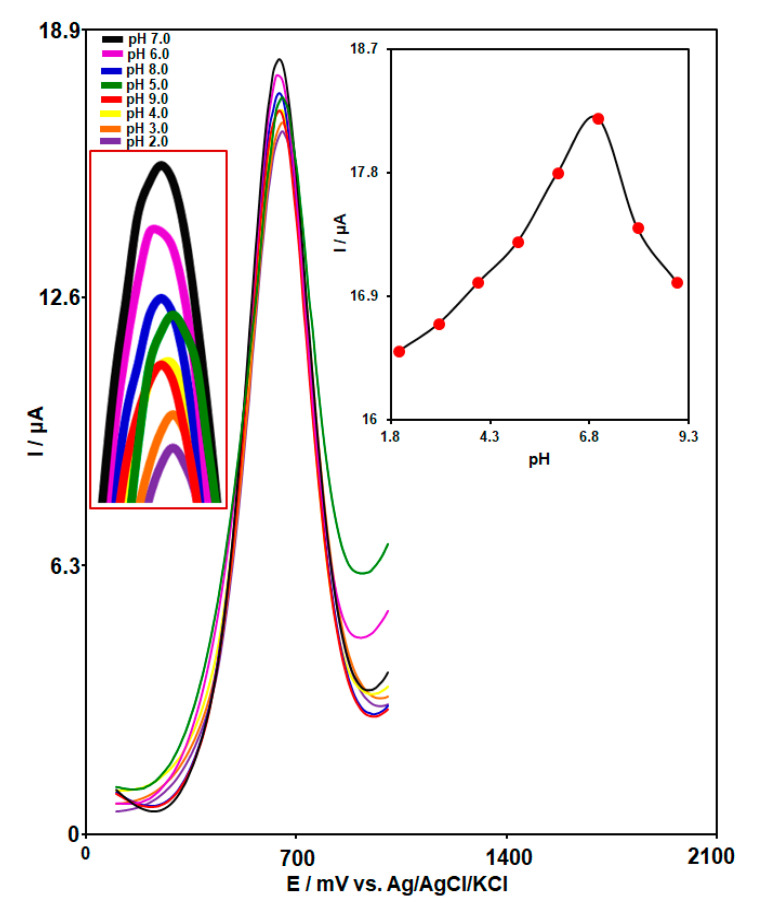
DPV curves of the MWCNT-Co_3_O_4_/BF/ILCPE in PBS (0.1 M) at various pH values (2.0, 3.0. 4.0, 5.0, 6.0, 7.0, 8.0 and 9.0) containing 50.0 µM D-PA. Inset: The plot of I_p_ against various pH solutions of 0.1 M PBS.

To examine the effect of the phosphate-buffered solution (PBS) pH on the electrochemical response of D-PA (50.0 µM), measurements were performed using DPV across the pH range 2.0 to 9.0 (Figure 1). The results on the MWCNT-Co_3_O_4_/BF/ILCPE surface showed that changing the pH of the buffer solution also changed the oxidation peak current (Ipa) of D-PA (Figure 1 (Inset)). The best Ipa for D-PA was obtained at pH 7.0. 

**Scheme 1:**
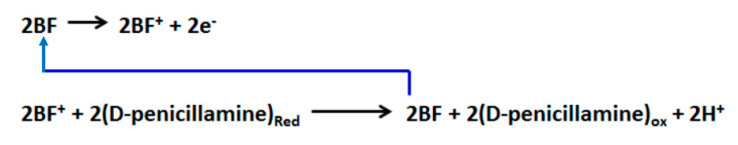
Electrocatalytic oxidation of D-PA at MWCNT-Co_3_O_4_/BF/ILCPE.

**Figure 2:**
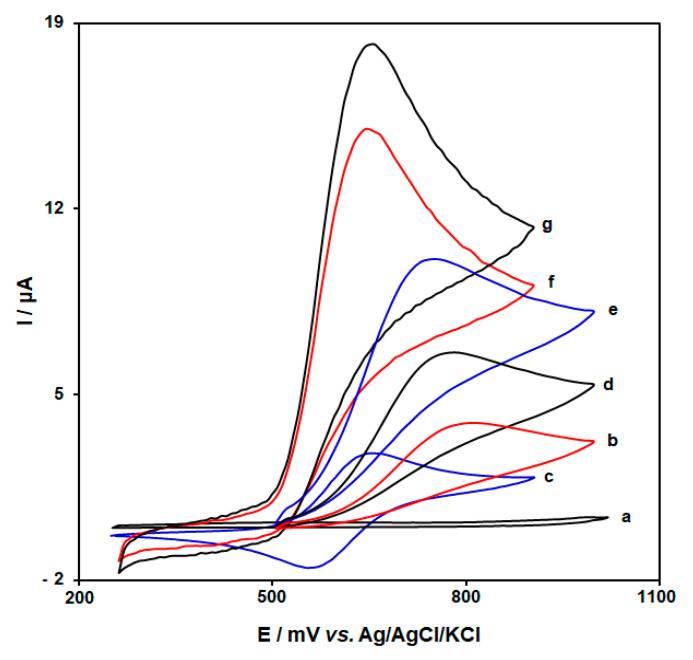
CV responses of: (a) un-modified CPE in 0.1 M PBS (pH = 7.0); (b) as (a) + 50.0 µM D-PA; (c) as (a) on the MWCNT-Co_3_O_4_/BF/ILCPE surface; (d) as (b) on the MWCNT-Co_3_O_4_/CPE surface; (e) as (b) on the MWCNT-Co_3_O_4_/ILCPE surface; (f) as (b) on the BF/ILCPE surface; and (g) as (b) on the MWCNT-Co_3_O_4_/BF/ILCPE surface. (In all cases, the scan rate was 10 mV s^−1^).

To investigate the electrochemical response of the D-PA (50.0 µM) on the surface of various electrodes, CV studies were performed. The corresponding recorded voltammograms (CVs) are shown in Figure 2. As can be seen, on the unmodified CPE in the presence of D-PA, the oxidation of D-PA occurs with a wide and weak peak at a potential of nearly 800 mV (voltammogram b), whereas no peak appears in the absence of D-PA (voltammogram a). After modifying the CPE using the MWCNT-Co_3_O_4_ nanocomposite in the presence of D-PA, the intensity of the oxidation current increased slightly and the oxidation potential was observed to be around 770 mV (voltammogram d). In the next step, the addition of the nanocomposite and IL to the composition of the CPE in the presence of D-PA led to an increase in current intensity and a decrease in potential (voltammogram e) compared to voltammogram d. Compared to the MWCNT-Co_3_O_4_/ILCPE, BF/ILCPE (voltammogram f) in the presence of D-PA clearly displayed a higher electrocatalytic activity towards D-PA (enhanced current intensity and reduced over-potential) due to the electrocatalytic activity of BF. On the surface of MWCNT-Co_3_O_4_/BF/ILCPE in the presence of D-PA, the anodic peak current that is observed for MWCNT-Co_3_O_4_/BF/ILCPE in the absence of D-PA (voltammogram c) increases greatly, while the corresponding cathodic peak disappears on the reverse scan (voltammogram g). The D-PA oxidation occurs at 650 mV at MMWCNT-Co_3_O_4_/BF/ILCPE surface; therefore, it is shifted ~150 mV towards a less positive potential compared to unmodified CPE. The oxidation current also significantly increased. From the results shown in Figure 2, the voltammetric response of D-PA on all the electrodes was evaluated and verified that the MWCNT-Co_3_O_4_/BF/ILCPE provides a significant improvement over other electrodes by increasing the current intensity and reducing the over-potential. These observations indicate that the electron transfer process on the MWCNT-Co_3_O_4_/BF/ILCPE has been facilitated, which can be attributed to the unique properties of the MWCNT-Co_3_O_4_ nanocomposite and IL, the electrocatalytic activity of BF, and their synergistic effects in the electron transfer process in D-PA oxidation. In addition, based on these findings, we propose a catalytic mechanism for the electrochemical oxidation of D-PA on MWCNT-Co_3_O_4_/BF/ILCPE, as illustrated in Scheme 1. According to the proposed mechanism, during the electrochemical reaction at the MWCNT-Co_3_O_4_/BF/ILCPE, an oxidized form of BF is generated and subsequently acts as a catalyst for the oxidation of D-PA [61].

### 3.2. Influence of Scan Rate 

**Figure 3:**
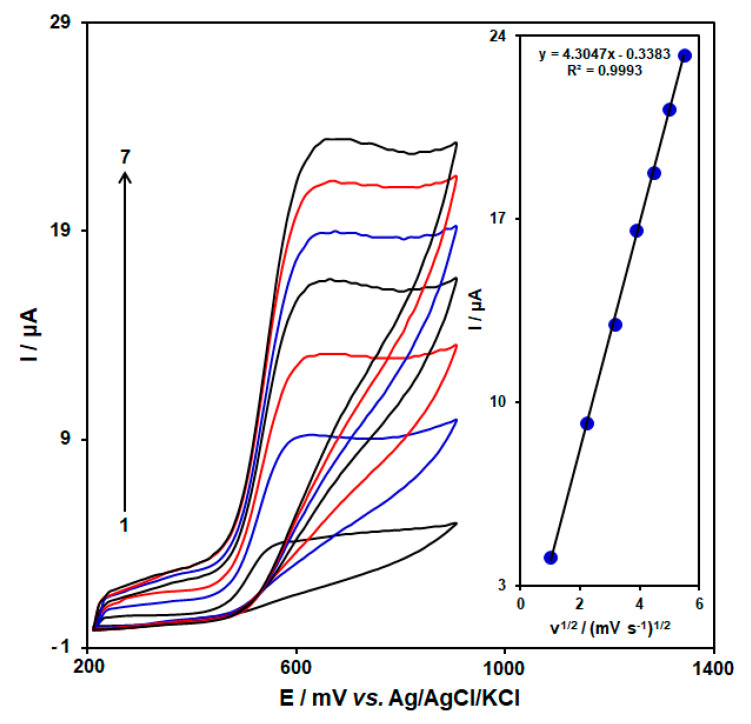
CVs observed for D-PA (20.0 μM) on MWCNT-Co_3_O_4_/BF/ILCPE in PBS (0.1 M, pH 7.0) at various scan rates (1: 1 mV s^−1^, 2: 5 mV s^−1^, 3: 10 mV s^−1^, 4: 15 mV s^−1^, 5: 20 mV s^−1^, 6: 25 mV s^−1^, and 7: 30 mV s^−1^). Inset: The linear plot of Ipa vs. ν^1/2^.

The influence of the scan rate on the electrochemical responses of D-PA on the MWCNT-Co_3_O_4_/BF/ILCPE was also assessed with the CV method by changing the scan rate from 1 to 30 mV·s^−1^ (Figure 3). It is apparent that the Ipa increases as the scan rate increases. From the observed CVs, a linear relationship was found between the electrocatalytic current of the D-PA and the square root of the scan rate ν^1/2^ (Inset of Figure 3). This finding demonstrates that the electrocatalytic oxidation of D-PA at MWCNT-Co_3_O_4_/BF/ILCPE is a diffusion-controlled process.

### 3.3. Chronoamperometric Analysis

**Figure 4:**
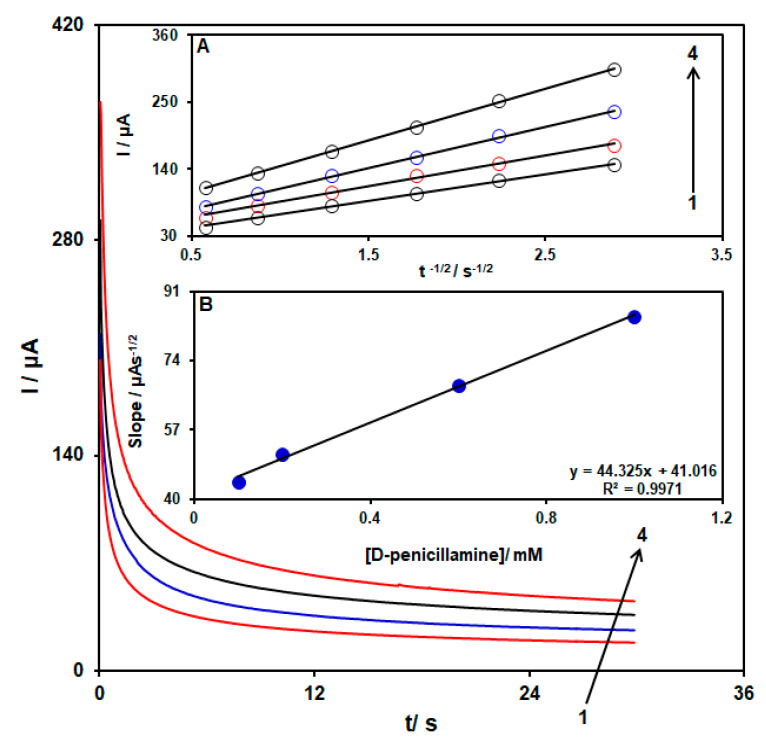
Chronoamperograms observed for D-PA with various concentrations (1: 0.1 mM, 2: 0.2 mM, 3: 0.6 mM, and 4: 1.0 mM) on MWCNT-Co_3_O_4_/BF/ILCPE in PBS (0.1 M, pH 7.0). Insets: (**A**) Variations of I vs. t^−1/2^ obtained from chronoamperograms 1–4, (**B**) Plot of the corresponding slopes vs. D-PA concentration.

The technique of chronoamperometry was utilized to determine the diffusion coefficient of an D-PA reaction. Figure 4 displays a set of graphs, known as chronoamperograms, that were obtained at a constant potential (700 mV) by utilizing MWCNT-Co_3_O_4_/BF/ILCPE in 0.1 M PBS (pH = 7.0) containing various D-PA concentrations. By using the Cottrell Equation (I = nFACD^1/2^π^−1/2^t^−1/2^), the diffusion coefficient (D) can be achieved by plotting the I versus t^−1/2^ (the plots of I vs. t^−1/2^ displayed straight lines for different concentrations of D-PA) (see Inset A, Figure 4), where D (cm^2^ s^−1^) is the diffusion coefficient; I (µA) is the current; C (mol cm^−3^) is the concentration; A (cm^2^) is the surface area of the electrode; F (96,485 C mol^−1^) is the Faraday’s constant; t (s) is the time; and n is the number of electrons transferred. Then, a curve can be plotted from a linear relationship between different D-PA concentrations (0.1 to 1.0 mM) and the obtained slopes from Inset A (see Inset B, Figure 4), leading to the calculation of a D value for D-PA (~8.2 × 10^−5^ cm^2^ s^−1^). The value of D is compared to 3.569 × 10^−6^ cm^2^ s^−1^ [16], 5.9 × 10^−4^ cm^2^ s^−1^ [18], 3.25 × 10^−5^ cm^2^ s^−1^ [20], 3.3 × 10^−5^ cm^2^ s^−1^ [22], and 2.5 × 10^−4^ cm^2^ s^−1^ [24].

### 3.4. Quantitative Analysis of D-PA by DPV

**Figure 5:**
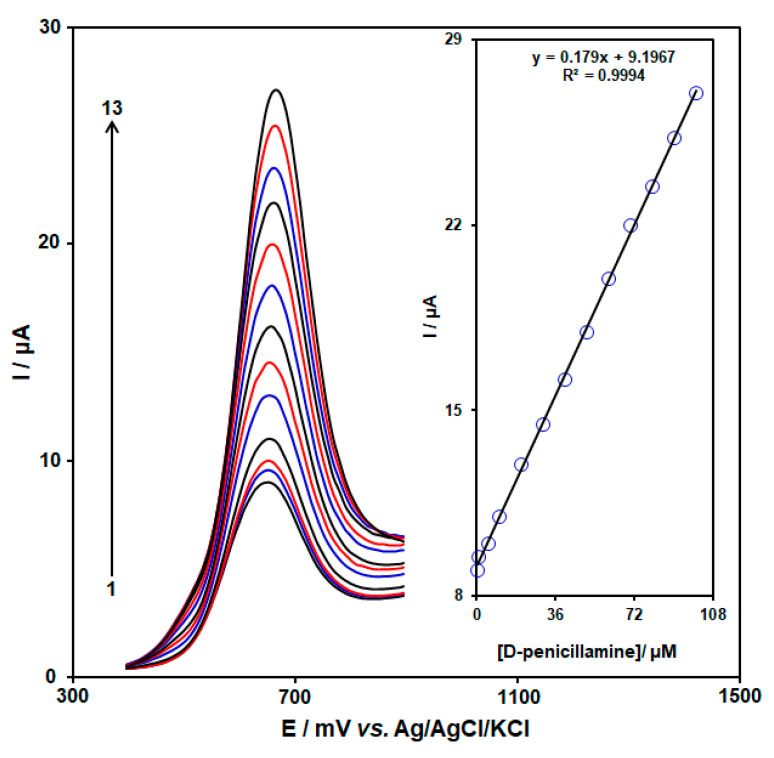
The DPVs observed for D-PA with concentrations varying from 0.05 µM to 100.0 µM (1: 0.05 µM, 2: 0.5 µM, 3: 5.0 µM, 4: 10.0 µM, 5: 20.0 µM, 6: 30.0 µM, 7: 40.0 µM, 8: 50.0 µM, 9: 60.0 µM, 10: 70.0 µM, 11: 80.0 µM, 12: 90.0 µM, and 13: 100.0 μM) on the MWCNT-Co_3_O_4_/BF/ILCPE in PBS (0.1 M, pH 7.0). Inset: Plot of anodic peak current of D-PA vs. its concentration.

The sensitivity of MWCNT-Co_3_O_4_/BF/ILCPE for quantitative analysis of D-PA under optimal conditions was assessed using DPV. The DPV responses of the developed CPE to various concentrations of D-PA in 0.1 M PBS (pH = 7.0) are illustrated in Figure 5. The observations indicated that the anodic peak current (Ipa) gradually increases with an increase in D-PA concentration. This observation pinpoints the remarkable performance of MWCNT-Co_3_O_4_/BF/ILCPE in the electrooxidation of D-PA. A plot between the concentration of D-PA and Ipa illustrates a linear relationship (Inset of Figure 5). From the linear plot and the corresponding linear regression equation Ipa (μA) = 0.179C_D-PA_ + 9.1967 (R^2^ = 0.9994), the detection limit (0.015 µM), linear range (0.05 μM to 100.0 μM), and sensitivity (0.179 μA/μM) were estimated.

The LOD was calculated using the following equation:

LOD = 3S_b_/m

where S_b_ and m represent the standard deviation of the response for the blank solution (PBS (0.1 M)) and the slope obtained from the linear regression curve, respectively.

An evaluation of the performance of the MWCNT-Co_3_O_4_/BF/ILCPE sensor to a number of electrochemical sensors reported in the literature for the determination of D-PA is presented in Table 1. It could be seen that the method developed based on MWCNT-Co_3_O_4_/BF/ILCPE provides the least LOD compared to the other reported works (Table 1). Thus, the present sensor with its good performance, fast and simple operation, and low-cost equipment can be an excellent tool for D-PA determination in pharmaceutical and biological samples.

### 3.5. Stability, Repeatability, and Reproducibility of MWCNT-Co_3_O_4_/BF/ILCPE for the Determination of D-PA

Essential requirements for designing and developing electrochemical sensors for practical applications include excellent repeatability, reproducibility, and long-term stability. The stability of the developed sensor was evaluated by examining the current response of the MWCNT-Co_3_O_4_/BF/ILCPE towards 50.0 µM D-PA every three days over 15 days (stored in ambient temperature). The developed sensor exhibited only a slight decrease (3.9%) in the last current response from its original current response after the 15-day storage. This finding evidences that the developed sensor has good storage stability. To investigate the repeatability of the MWCNT-Co_3_O_4_/BF/ILCPE sensor, the measurements were repeated in 0.1 M PBS (pH = 7.0) containing 50.0 µM D-PA. Acceptable repeatability was obtained with RSD of 3.2% after using the same sensor for five continuous measurements. The reproducibility of the developed sensor was also evaluated by recording the current response of five electrodes prepared independently under the same conditions. All five prepared sensors showed similar responses and the RSD was 4.2% in the determination of D-PA. The obtained results verify the acceptable reproducibility of the MWCNT-Co_3_O_4_/BF/ILCPE for D-PA sensing.

### 3.6. D-PA Analysis in D-PA Capsules and Urine Samples

**Figure 6:**
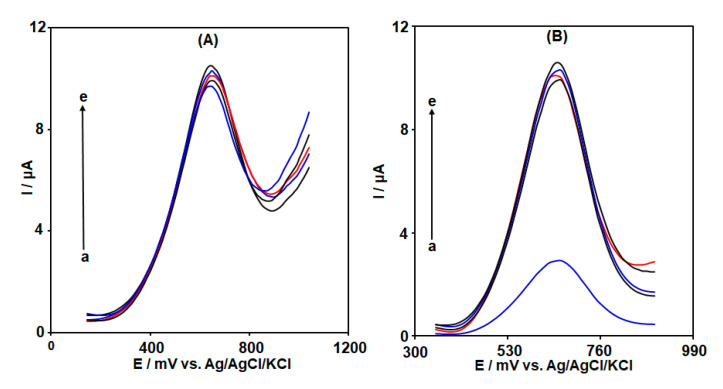
Typical DPVs obtained at MWCNT-Co_3_O_4_/BF/ILCPE in 0.1 M PBS (pH = 7.0) for analysis of real samples: (**A**) D-PA capsules spiked with different concentrations of D-PA (a: 0, b: 1.0, c: 2.0, d: 3.0, and e: 4.0 μM) and (**B**) urine samples spiked with different concentrations of D-PA (a: 0, b: 5.0, c: 6.0, d: 7.0, and e: 8.0 μM).

The MWCNT-Co_3_O_4_/BF/ILCPE was applied to detect D-PA in D-PA capsules and urine to demonstrate its practical applicability. The contents of D-PA in samples were detected by the standard addition method. The obtained typical voltammograms are shown in Figure 6. Table 2 displays the obtained results. The acceptable recoveries (between 96.7% and 104.3%) and low values of RSD (between 1.8% and 3.4%) were obtained, which suggests that the method used for detection is reliable and accurate.

## 4. Conclusions

The present attempt was made to prepare a new electrochemical sensor based on the modification of CPE using MWCNT-Co_3_O_4_nanocomoisite, BF, and IL for electrocatalytically sensing D-penicillamine. The MWCNT-Co_3_O_4_ nanocomposite, IL, and BF produced a voltammetric sensor with a large conductive surface area capable of accelerating electron transfer and current signal amplification. This sensor displayed an admirable electrocatalytic ability towards D-PA oxidation. The developed MWCNT-Co_3_O_4_/BF/ILCPE sensor exhibited a high sensitivity of 0.179 µA/µM with a wide linear range from 0.05 μM to 100.0 M, and a detection limit of 0.015 µM. Also, the MWCNT-Co_3_O_4_/BF/ILCPE sensor displayed acceptable repeatability (five consecutive measurements with RSD of 3.2%), reproducibility (five independent electrodes with RSD of 4.2%) and long-term stability (with a 3.9% decrease from its original current response after 15 days). According to the results from a practical assessment of the MWCNT-Co_3_O_4_/BF/ILCPE using spiked samples, the sensor showed an acceptable recovery percentage range of 96.7–104.3% and RSD values ≤ 3.4.

## Figures and Tables

**Table 1:** Comparison of the performance of the MWCNT-Co_3_O_4_/BF/ILCPE sensor with other reported works for D-PA determination.

Electrochemical Sensor	Electrochemical Method	Linear Range	Limit of Detection	Ref.
Polydiphenylamine@electrochemically reduced graphene oxide/glassy carbon electrode	Amperometry	1.4 μM–541 µM	0.10 µM	[5]
Ni_3_S_4_/NiS_2_/MoOx composite/glassy carbon electrode	Amperometry	5 μM–796 μM	0.26 µM	[16]
Au nanoparticle-reduced graphene oxide/glassy carbon electrode	DPV	5 μM–110 μM	3.9 µM	[17]
Multi-walled carbon nanotubes/CPE in the presence of methyldopa as a mediator	Square wave voltammetry	0.2 µM–250.0 µM	0.1 µM	[18]
Potassium iodide (mediator)/glassy carbon electrode	DPV	9 µM–120 µM	3.5 µM	[19]
	CV	30 µM–1500 µM	30 µM	
Catechol (electrochemical indicator)/CPE	DPV	70 µM–1000 µM	50 µM	[20]
	CV	100 µM–1000 µM	58 µM	
Ferrocene carboxylic acid/CPE	DPV	6.5 µM–100 µM	6.15 µM	[21]
	CV	75 µM–1000 µM	60.4 µM	
ZnIn_2_S_4_ nanoparticles/CPE	Square wave voltammetry	0.5 μM–80.0 μM	0.3 µM	[22]
Cobalt salophen Schiff base complex/CPE	Square wave voltammetry	0.1 µM–100.0 µM	0.1 µM	[23]
Multi-walled carbon nanotubes paste electrode in the presence of chlorpromazine as a mediator	Linear sweep voltammetry	0.5 μM–500 μM	0.2 µM	[24]
MWCNT-Co_3_O_4_/BF/ILCPE	DPV	0.05–100.0 μM	0.015 µM	This work

**Table 2:** Voltammetric determination of D-PA in D-PA capsules and urine samples using MWCNT-Co_3_O_4_/BF/ILCPE (n = 5).

Sample	Spiked Concentration	Found Concentration	Recovery	R.S.D. (%)
D-PA capsules	0	3.7 μM	-	3.4%
	1.0 μM	4.6 μM	97.9%	1.9%
	2.0 μM	5.9 μM	103.5%	2.4%
	3.0 μM	6.8 μM	101.5%	3.0%
	4.0 μM	7.6 μM	98.7%	2.7%
Urine	0	-	-	-
	5.0 μM	5.1 μM	102.0%	1.8%
	6.0 μM	5.8 μM	96.7%	3.4%
	7.0 μM	7.3 μM	104.3%	2.3%
	8.0 μM	7.9 μM	98.7%	2.1%

## Data Availability

The data presented in this study are available on request from the corresponding authors.
